# The Action of Medicines

**Published:** 1878-01

**Authors:** 


THE ACTION OF MEDICINES. By Isaac Ott, A.M., M.D, formerly Demonstrator of Experimental Physiology, University of Pennsylvania. With twenty-two illustrations. Philadelphia: Lindsay & Blakiston, 1878.

This octavo volume of 164 pages contains more useful instruction than can be found in half a dozen large volumes of new publications we have seen. The author seeks to promote advancement in therapeutics by a more thorough knowledge of the physiological action of remedies. This is certainly the correct method, and his efforts will be appreciated by the student of acology and therapeutics.

The time has passed for the books of routinists to be viewed as undoubted authority for the administration of this and that medicine for the cure of particular diseases, without an intimation of the action produced by the remedy. The time of formularies and recipes is passing away. They are not now acceptable to correct medical thinkers.

In the work before us the author gives instruction in experimental study of the physiological action of medicines on inferior animals; in the physiology of the nervous and circulatory systems, and in the comparative physiological action of remedies on man and inferior animals, with their therapeutic results in disease.

While the work does not form a complete text-book for the action of all remedies in use, the author has accomplished all he proposed, and has furnished a book which should be read and studied by all lovers of rational medicine.

A thorough text-book on materia medica and therapeutics must necessarily contain all the medical substances in common use, whose physiological action is known, and a description of the action had by a medicinal dose. This course has not been pursued by the author, nor was it his purpose to do more than he has accomplished.

